# Early Oral Immunotherapy With Pasteurized Egg White in Egg‐Allergic Children Under 2 Years of Age

**DOI:** 10.1002/clt2.70155

**Published:** 2026-02-26

**Authors:** Silvia Karina Carrión Sari, Luis Martínez‐Lostao, Carlos Colás Sanz, María Teresa Sobrevía Elfau

**Affiliations:** ^1^ Unidad de Alergología Hospital Santa Bárbara Soria Spain; ^2^ Servicio de Immunología Hospital Clínico Lozano Blesa Zaragoza Spain; ^3^ Instituto de Investigación Sanitaria de Aragón Zaragoza Spain; ^4^ Facultad de Medicina Universidad de Zaragoza Zaragoza Spain; ^5^ Servicio de Alergología Hospital Clínico Lozano Blesa Zaragoza Spain

To the Editor:

In Europe, the overall raw incidence of egg allergy in the first 2 years of life is 0.84% (0.78% in Spain) [1]. Few publications are available on the safety of oral immunotherapy (OIT) in young children [2, 3, 4].

Our objective was to assess the possibility of desensitization after OIT with pasteurized egg white in patients under 2 years of age with egg allergy. This was a prospective observational analytical study in which children under 2 years of age with IgE‐mediated egg allergy were recruited and received OIT with pasteurized liquid egg white (Huevos Guillén S.L.).

Children attending the Allergology Department of the Hospital Clínico Lozano Blesa in Zaragoza, Spain, were recruited. All patients who met the inclusion criteria were included consecutively. The study was approved by the Research Ethics Committee of Autonomous Community of Aragón and informed consent was obtained from both parents.

Inclusion criteria were history of immediate reaction (< 1h) after ingestion of egg; IgE > 0.35 kU/L and/or skin prick test (SPT) wheal ≥ 3 mm to at least 1 egg fraction (egg white, yolk, ovalbumin or ovomucoid); and a positive oral food challenge (OFC) with boiled egg. OFC was performed on all patients except those with a history of anaphylactic reaction (diagnosed according to Sampson's criteria [5]) or ≥ 2 immediate reactions in the last 3 months. SPT and determinations of specific IgE (sIgE), specific IgG4 (sIgG4), and sIgE/total IgE ratio were performed at T1 (baseline), at T2 (end of dose escalation phase), and at T3 (6 months after T2).

During the induction phase, increasing doses of pasteurized egg white were administered weekly in the hospital (Supporting Information S1: Table S1). The final dose was 30 mL of egg white (3300 mg of protein, the amount of egg white in a medium‐sized egg). Between the increments, the dose tolerated in the clinic was repeated daily at home until the next visit. An OFC with natural raw egg was performed during the T2 visit to verify total desensitization. The maintenance phase, lasting 6 months, consisted of administration of the total dose (30 mL) on alternate days for a period of 15 days, followed by ingestion of 1 egg prepared in different ways (boiled, scrambled, baked, etc.), including undercooked preparations (mayonnaise, mousse, etc.) at least 3 times a week, until the T3 follow‐up visit. Adherence was monitored by parental reporting.

A total of 31 patients were recruited, 17 (54.8%) females and 14 (45.2%) males. Mean age at presentation of the first symptom was 9.6 months (SD 2.48). Median age at the start of OIT was 14 months (range 9–26). Median age at diagnosis was 11 months (IQR: 3.71). Fifteen (48.39%) patients had a history of atopic dermatitis; 11 (35.48%) patients had previously presented wheezing. Eleven (35.48%) patients had a history of other food allergies, and 15 (53.57%) had a family history of allergic diseases.

Statistically significant decreases (*p* ≤ 0.001) in SPT wheal diameter, sIgE levels, and sIgE/total IgE ratio were observed (Table 1). Higher sIgG4 was seen at T2, followed by a slight decline at T3 (Figure 1), although no significant differences were observed.

**TABLE 1:** Immunological findings at baseline and after 6 months of observation.

Variable (*n* = 31)		T1	T3	*p*‐value
Wheal diameter of EW SPT	Median (range) mm	6.00 (0–16)	0.00 (0–9)	< 0.001
Wheal diameter of EY SPT	Median (range) mm	4.00 (0–14)	0.00 (0–7)	< 0.001
Wheal diameter of OVA SPT	Median (range) mm	7.00 (0–14)	0.00 (0–8)	< 0.001
Wheal diameter of OVM SPT	Median (range) mm	8.00 (0–20)	0.00 (0–10)	< 0.001
Total IgE	Median (range) kU/L	25.20 (3.94–868)	79.65 (3.94–3460)	0.013
EW sIgE	Median (range) kU/L	1.95 (0.16–51.30)	0.34 (0–7.08)	< 0.001
EY sIgE	Median (range) kU/L	0.45 (0–11.90)	0.00 (0–2.78)	< 0.001
OVA sIgE	Median (range) kU/L	0.73 (0–52.80)	0.16 (0–8.68)	< 0.001
OVM sIgE	Median (range) kU/L	1.80 (0–55.70)	0.25 (0–3.71)	< 0.001
EW sIgG4	Median (range) mg/L	0.12 (0.01–0.65)	16.80 (0.2–30)	0.109
EY sIgG4	Median (range) mg/L	0.10 (0.06–0.38)	11.10 (0.09–30)	< 0.001
OVA sIgG4	Median (range) mg/L	0.05 (0.00–0.40)	16.70 (0.06–30)	0.109
OVM sIgG4	Median (range) mg/L	0.03 (0.00–0.90)	6.31 (0.12–30)	0.180
EW sIgE/total IgE ratio	Median (range) kU/L	0.06 (0.01–0.32)	0.00 (0–0.09)	< 0.001
EY sIgE/total IgE ratio	Median (range) kU/L	0.01 (0–0.13)	0.00 (0–0.01)	< 0.001
OVA sIgE/total IgE ratio	Median (range) kU/L	0.03 (0–0.22)	0.00 (0–0.09)	< 0.001
OVM sIgE/total IgE ratio	Median (range) kU/L	0.05 (0–0.56)	0.00 (0–0.03)	< 0.001

*Note:* Wilcoxon signed‐rank test.

Abbreviations: EG: Egg white; EY: Egg yolk; OVA: ovalbumin; OVM: ovomucoid; SPT: skin prick test; T1: baseline; T3: 6 months after T2 (maintenance phase).

**FIGURE 1:**
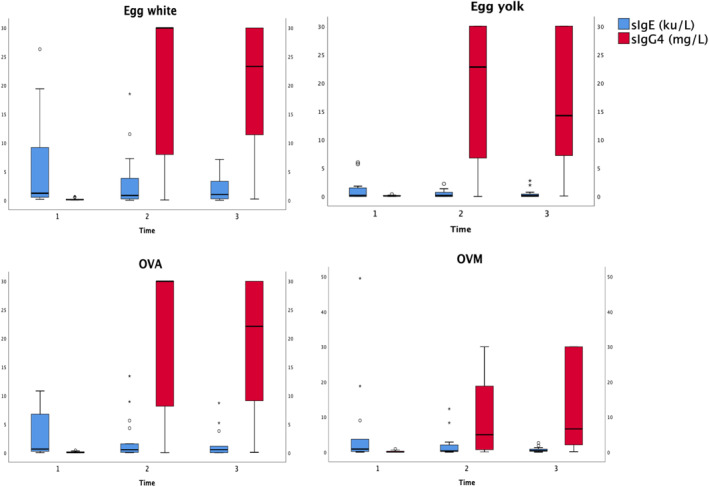
Progress over time of specific IgE and specific IgG4 to egg white, egg yolk, ovoalbumin and ovomucoid. OVA: ovalbumin; OVM: ovomucoid; sIgE: specific IgE antibody levels; T1: baseline; T2: end of induction phase; T3: 6 months after T2 (maintenance phase).

During the induction phase, 24 patients (77.42%) had adverse reactions: 71 (65.14%) mild skin reactions; 31 (28.44%) gastrointestinal effects; and 7 (6.42%) rhinoconjunctivitis. Most events resolved spontaneously or with oral antihistamines. None required adrenaline. OFC was negative in 30 patients at T2. One patient, who reached an OIT dose of 22 mL, discontinued the study for family reasons, and at 6 months could tolerate only food containing baked egg and small amounts of boiled egg. Another patient with a negative OFC subsequently refused the maintenance ingestions and accepted only baked egg and egg traces. Six patients (19.35%) developed reactions in the maintenance phase: 4 cutaneous and 2 gastrointestinal. All these reactions were mild and did not prevent the patients from subsequently continuing to consume eggs.

Giavi [2] reported a 36% rate of negative OFCs at 6 months. In our study, during follow‐up, 93.54% (29/31) of the parents reported that their children remained in a state of desensitization, so OFC was not performed. The proportion achieving complete desensitization (absence of reaction to a medium‐sized egg, ranging from raw to thoroughly cooked) was higher than in previous studies (64%–84%) [4, 6]. OIT is not risk‐free, but our rate of adverse reactions is in line with published systematic reviews [7]. None of our patients needed adrenaline, whereas in a study in older children conducted by Vazquez‐Ortiz et al. [8], it was required in 26% of 50 children aged 5–18 years. Immunological changes after OIT similar to our series have been previously described in several studies [2, 3, 4]. One strength of our study is the young age of our patients: recent trials with peanuts and milk show that early intervention improves the outcomes of OIT [9]. Another is the use of pasteurized egg white, selected for this study for its stability, reproducibility, allergenicity, ease of dosing, low cost, and microbiological safety.

Our study is limited by the relatively short follow‐up period without assessment of long‐term tolerance, the lack of randomization, and the potential compromise of statistical inference due to the absence of a control group. There is also a potential for selection bias (e.g., families were highly motivated to pursue OIT in a tertiary center).

Although the results of our series seem to indicate that early oral immunotherapy is effective for the development of egg desensitization, we believe that further controlled and randomized studies are required to confirm this statement.

## Author Contributions

**Silvia Karina Carrión Sari:** funding acquisition, investigation, formal analysis, writing – original draft, visualization, software, data curation. **Luis Martínez‐Lostao:** writing – review and editing, supervision, investigation, project administration. **Carlos Colás Sanz:** supervision, methodology, writing – review and editing, conceptualization, investigation. **María Teresa Sobrevía Elfau:** conceptualization, methodology, investigation, writing – review and editing, supervision, validation.

## Funding

This study has been funded by the SEAIC (Spanish Society of Allergology and Clinical Immunology) Foundation Ref: 22A12.

## Conflicts of Interest

The authors declare no conflicts of interest.

## Supporting information


Supporting Information S1

## Data Availability

The data that support the findings of this study are available from the corresponding author upon reasonable request.

## References

[1] P. Xepapadaki , A. Fiocchi , L. Grabenhenrich , et al., “Incidence and Natural History of Hen's Egg Allergy in the First 2 Years of Life‐the EuroPrevall Birth Cohort Study,” Allergy 71, no. 3 (2016): 350–357, 10.1111/all.12801. 26514330

[2] S. Giavi , Y. M. Vissers , A. Muraro , et al., “Oral Immunotherapy With Low Allergenic Hydrolysed Egg in Egg Allergic Children,” Allergy 71, no. 11 (2016): 1575–1584, 10.1111/all.12905. 27059671

[3] A. D. Buchanan , T. D. Green , S. M. Jones , et al., “Egg Oral Immunotherapy in Nonanaphylactic Children With Egg Allergy,” Journal of Allergy and Clinical Immunology 119, no. 1 (2007): 199–205, 10.1016/j.jaci.2006.09.016. 17208602

[4] U. Staden , C. Rolinck‐Werninghaus , F. Brewe , U. Wahn , B. Niggemann , and K. Beyer , “Specific Oral Tolerance Induction in Food Allergy in Children: Efficacy and Clinical Patterns of Reaction,” Allergy 62, no. 11 (2007): 1261–1269, 10.1111/j.1398-9995.2007.01501.x. 17919140

[5] H. A. Sampson , A. Munoz‐Furlong , R. L. Campbell , et al., “Second Symposium on the Definition and Management of Anaphylaxis: Summary Report‐‐Second National Institute of Allergy and Infectious Disease/Food Allergy and Anaphylaxis Network Symposium,” Journal of Allergy and Clinical Immunology 117, no. 2 (2006): 391–397, 10.1016/j.jaci.2005.12.1303. 16461139

[6] M. F. Martin‐Munoz , M. T. Belver , E. Alonso Lebrero , et al., “Egg Oral Immunotherapy in Children (SEICAP I): Daily or Weekly Desensitization Pattern,” Pediatric Allergy & Immunology 30, no. 1 (2018): 81–92, 10.1111/pai.12974. 30169915

[7] O. Romantsik , M. Bruschettini , M. A. Tosca , S. Zappettini , O. Della Casa Alberighi , and M. G. Calevo , “Oral and Sublingual Immunotherapy for Egg Allergy,” Cochrane Database of Systematic Reviews 4, no. 11 (2014): CD010638, 10.1002/14651858.CD010638.pub3. 25405335

[8] M. Vazquez‐Ortiz , M. Alvaro , M. Piquer , et al., “Baseline Specific IgE Levels Are Useful to Predict Safety of Oral Immunotherapy in Egg‐Allergic Children,” Clinical and Experimental Allergy 44, no. 1 (2014): 130–141, 10.1111/cea.12233. 24355019

[9] L. J. C. Barten , M. Zuurveld , J. Faber , J. Garssen , and T. Klok , “Oral Immunotherapy as a Curative Treatment for Food‐Allergic Preschool Children: Current Evidence and Potential Underlying Mechanisms,” Pediatric Allergy & Immunology 34, no. 11 (2023): e14043, 10.1111/pai.14043. 38010006

