# Supplementary material for: Early Oral Immunotherapy With Pasteurized Egg White in Egg‐Allergic Children Under 2 Years of Age
Source: Clin Transl Allergy. 2026 Feb 26;16(3):e70155. doi: 10.1002/clt2.70155 (PMC12945664; doi:10.1002/clt2.70155)
Supplement: Supplementary file 1 — Supporting Information S1 [file CLT2-16-e70155-s001.docx]

**Supplementary material**

**Immunologic findings and mechanisms of desensitization.**

OIT leads to an increase of suppressor cytokine IL-10 and IFN-α production by DCs and subsequent Th2 anergy and an increase in regulatory cells, including Tregs. This results in decreased production of sIgE and increased production of sIgG, including sIgG4, and sIgA. These changes lead to downstream suppression of the allergic response.(1)

Since some patients have elevated levels of total IgE, because food allergy is often associated with other atopic diseases, the specific IgE/total IgE ratio has been used for a more accurate interpretation.(1) sIgE and sIgG4 measurements were done using ImmunoCAP platform.

**Additional data**

The severity of disease at baseline were: 6 patients (20%) were Clark grade 1; 8 (26.66%) were grade 2; 15 (50%) were grade 3; and 1 (3.33%) was grade 4 (2).

The median dose of cooked egg that produced symptoms in patients during the baseline cooked egg challenge was 8.70 g (range: 1.56-25 g). The mean age at the time of the OFC was 12.49 months (range: 8.7- 20.33 months).

The symptoms experienced by the patients during the cooked egg screening OFC were: urticaria-angioedema (57.14%); vomiting (14.28%); and anaphylaxis (28.57%). Adrenaline was used in all patients who presented anaphylaxis.

The median age at which patients began egg OIT was 14 months, range 9-26 months. The start of OIT was delayed in 2 patients due to the COVID pandemic. The mean duration of OIT induction phase was 167.3 days, (5.58 months), SD: 44.78.

The OFC with natural raw egg at T2 was performed upon completing induction phase Total desensitization was verified. The mean time from the OFC at T1 to the OFC at T2 was 6.77 months, SD: 8.3. **Standard microbiological practices were used for OFC. These included:** proper handwashing, disinfection of surfaces, keeping eggs refrigerated at 5°C or colder to slow down bacterial growth, discarding any eggs with cracks in the shell, and always using the eggs before expiration date.

**Table 1. ORAL IMMUNOTHERAPY PROTOCOL WITH PASTEURIZED EGG WHITE**

| **Week no.** | **Dilution** | **Single dose (ml)** | **Protein dose (mg)** |
| --- | --- | --- | --- |
| **1** | 1/1 | 0,1 | 11 |
| **2** | 1/1 | 0,2 | 22 |
| **3** | 1/1 | 0,4 | 44 |
| **4** | 1/1 | 0,7 | 77 |
| **5** | 1/1 | 1 | 110 |
| **6** | 1/1 | 1,3 | 143 |
| **7** | 1/1 | 2 | 220 |
| **8** | 1/1 | 2,5 | 275 |
| **9** | 1/1 | 3,2 | 352 |
| **10** | 1/1 | 4 | 440 |
| **11** | 1/1 | 5 | 550 |
| **12** | 1/1 | 6,2 | 682 |
| **13** | 1/1 | 8 | 880 |
| **14** | 1/1 | 11 | 1210 |
| **15** | 1/1 | 15 | 1650 |
| **16** | 1/1 | 22 | 2475 |
| **17** | 1/1 | 30 | 3300 |

**References:**

1. Barten LJC, Zuurveld M, Faber J, Garssen J, Klok T. Oral immunotherapy as a curative treatment for food-allergic preschool children: Current evidence and potential underlying mechanisms. Pediatr Allergy Immunol. 2023;34(11):e14043.

2. Clark AT, Ewan P W. Food allergy in childhood. Arch Dis Child. 2003;88(1):79-81.
